# Sleep disorders in Down syndrome: a systematic review

**DOI:** 10.1590/0004-282X-ANP-2021-0242

**Published:** 2022-03-14

**Authors:** Ravenna Araújo Santos, Lellis Henrique Costa, Rebeca Coêlho Linhares, Márcia Pradella-Hallinan, Fernando Morgadinho Santos Coelho, Giuliano da Paz Oliveira

**Affiliations:** 1 Universidade Federal Delta do Parnaíba, Parnaíba PI, Brazil.; 2 Faculdade de Ciências Humanas, Exatas e de Saúde do Piauí, Instituto de Educação Superior do Vale do Parnaíba, Parnaíba PI, Brazil.; 3 Universidade Federal de São Paulo, Departamento de Psicobiologia, São Paulo SP, Brazil.; 4 Universidade Federal de São Paulo, Departamento de Neurologia e Neurocirurgia, São Paulo SP, Brazil.

**Keywords:** Down Syndrome, Sleep Wake Disorders, Sleep Apnea Syndromes, Síndrome de Down, Transtornos do Sono-Vigília, Síndromes da Apneia do Sono

## Abstract

**Background:**

Sleep disorders are commonly observed in children with Down syndrome (DS) and can lead to significant behavioral and cognitive morbidities in these individuals.

**Objective:**

To perform a systematic review evaluating sleep disorders in individuals with DS.

**Methods:**

Search strategies were based on combinations of keywords: “Down syndrome”; “trisomy 21”; “sleep disorders”; “dyssomnias”; “sleep apnea”; “obstructive”; “sleeplessness”; “insomnia”; “parasomnias”; and “excessive daytime sleepiness”. PubMed and Science Direct were used. Only original studies and retrospective reviews in English published between January 2011 and March 2021 were included.

**Results:**

52 articles were included, most of them involving children and adolescents under 18 years of age. The main sleep disorder associated with DS was obstructive sleep apnea (OSA). Some studies reported the presence of cognitive dysfunction in patients with DS and sleep-disordered breathing, and few have been found about parasomnia, insomnia, and daytime sleepiness in these patients. Movement disorders and unusual postures during sleep may be related to disordered sleep breathing in DS. The main treatment options for OSA are continuous positive airway pressure therapy (CPAP), surgery, and weight control. Computational modeling associated with MRI has been used to plan surgical interventions in these patients.

**Conclusions:**

Individuals with DS are at high risk of developing sleep-related breathing disorders. The main sleep disorder associated with DS was OSA. The presence of sleep-disordered breathing contributes to a worsening of cognitive function in patients with DS.

## RESUMO

**Antecedentes:**

Os distúrbios do sono são comumente observados em crianças com síndrome de Down (SD) e podem levar a morbidades comportamentais e cognitivas significativas nesses indivíduos.

**Objetivo:**

Realizar uma revisão sistemática para avaliar os distúrbios do sono em indivíduos com SD.

**Métodos:**

As estratégias de busca foram baseadas em combinações de palavras-chave: “Síndrome de Down”; “trissomia 21”; “distúrbios do sono”; “dissonias”; “apneia do sono”; “obstrutivo”; “insônia”; “insônia”; “parassonias” e “sonolência diurna excessiva”. PubMed e Science Direct foram usados. Apenas estudos originais e revisão retrospectiva de prontuários escritos em inglês e publicados de janeiro de 2011 a março de 2021 foram incluídos.

**Resultados:**

Foram selecionados 52 artigos, a maioria com crianças e adolescentes menores de 18 anos. O principal distúrbio do sono associado à SD foi a apneia obstrutiva do sono (AOS). Alguns estudos relatam a presença de disfunção cognitiva em pacientes com SD e distúrbios respiratórios do sono, e poucos foram encontrados sobre parassonia, insônia e sonolência diurna nesses pacientes. Distúrbios do movimento e posturas incomuns durante o sono podem estar relacionados ao distúrbio respiratório do sono na SD. As principais opções de tratamento para AOS são pressão positiva contínua nas vias aéreas (CPAP), abordagem cirúrgica e controle de peso. A modelagem computacional associada à ressonância magnética tem sido usada para planejar intervenções cirúrgicas nesses pacientes.

**Conclusões:**

Indivíduos com SD apresentam alto risco de desenvolver distúrbios respiratórios relacionados ao sono. O principal distúrbio do sono associado à SD foi a AOS. A presença de distúrbios respiratórios do sono contribui para a piora das funções cognitivas em pacientes com SD.

## INTRODUCTION

Down syndrome (DS) was first characterized in 1866 by John Langdon Down, who described “individuals with peculiar clinical manifestations”. Furthermore, in 1958, Jérôme Lejeune and Pat Jacobs stated that DS is a genetic syndrome related to a trisomy of chromosome 21. The DS prevalence in USA is around 13.56 for every 10,000 live births^1–3^.

Clinical manifestations vary widely from person to person, but cognitive impairment is commonly noted in this syndrome^4,5^. Also, there are some common phenotypic features in individuals with DS, such as muscle hypotonia, macroglossia, brachycephaly, epicanthal folds, flat nasal bridge, micrognathia, low-set ears, excessive skin on the nape, single transverse palmar crease, clinodactyly of the fifth finger, and a larger gap between the first and second toes^6,7^.

Sleep plays a critical role in good health and well-being. For this reason, sleep disorders in children and adolescents are associated with problems in physical, behavioral, and physiological development and pose an additional risk for obesity, endocrine disorders, depression, immunological, and heart diseases^8–10^. These disorders are commonly observed in children with DS and can lead to significant behavioral and cognitive morbidities in individuals with DS^11–13^.

The aim of this study was to provide a systematic review to evaluate sleep disorders in people with Down syndrome, focusing on clinical presentation, pathophysiology, and treatment strategies.

## METHODS

A systematic review of the literature, based on the PRISMA statement and the recommendations for systematic review and meta-analysis, was conducted to investigate the main sleep disorders in patients with Down syndrome and their treatment^13^. Search strategies were based on combinations of keywords “Down syndrome”, “trisomy 21”, “sleep disorders”, “dyssomnias”, “sleep apnea”, “obstructive”, “sleeplessness”, “insomnia”, “parasomnias”, and “excessive daytime sleepiness”, which were defined based on previous research in the Medical Subject Headings (MeSH) system. PubMed and Science Direct were used as databases, with a publication period of January 2011 to March 2021.

Researchers 1 and 2 (R.A.S and L.H.C) considered the topics covered in each article searched, in addition to the inclusion and exclusion criteria. Treatment-only studies were excluded; the focus was on studies that addressed sleep disorders in patients with DS.

Inclusion criteria were: original studies and retrospective chart reviews written in in English and with no restriction on health, age, or gender of subjects. Exclusion criteria were: papers not related to sleep disorders in DS patients after reading the full text and editorials, letters to the editor, review articles, case reports, and meeting abstracts. The collected data were compiled into a spreadsheet containing all relevant information from the studies, including authors, year of publication, journal name, sample characteristics (size, gender, age, and geographic area), data collection methods, clinical diagnosis, and assessed sleep disorder.

## RESULTS

**Figure 1:**
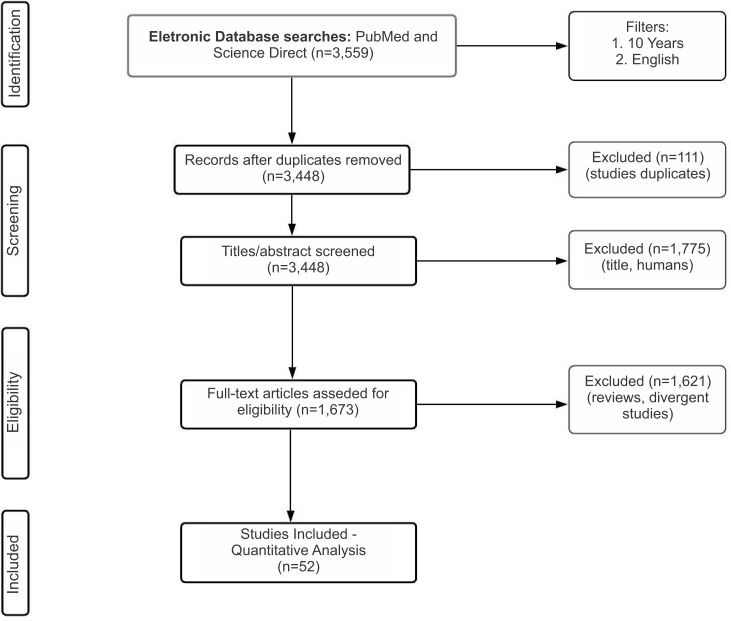
Flowchart of the literature search based on the recommendations of the Preferred Reporting Items for Systematic Reviews and Meta-Analysis.

An initial search identified 3559 studies from the past 10 years. Subsequently, editorials, letters to the editor, review articles, case reports, meeting abstracts, and laboratory-based studies, including animal studies, were excluded, remaining 163 articles. After reading full-text articles, that met all predefined criteria, and excluding duplicates, 52 articles were included in this systematic review (Figure 1).

Among the selected studies were papers from 14 countries, most of them from the USA and Belgium. Regarding the population studied, most studies included children and adolescents under 18 years of age, and only 9 included the adult population. The main results are summarized in Tables 1 to 4. Almost all studies were case series, and about 50% of the manuscripts used PSG to define OSA.

**Table 1:** Synthesis of articles selected for systematic review on prevalence, etiology, correlating factors, screening methods, and biomarkers for obstructive sleep apnea in Down syndrome patients (age>18 years).

Author/year	Study	N° of patients	Mean age (years/months)	Time of follow up	Outcome	Conclusion
Carvalho et al., 2020^49^	Case series-Questionnaires; blood county	60 (DS)	>18 years	-	Adults with DS have a very high prevalence of OSA. Hematocrit levels, STOP-Bang questionnaires (SBQ) showed a strong correlation with OSA severity. The SBQ performed well in identifying moderate to severe OSA in this population.	Considered together, these results point to the need to perform OSA screening in all adults with DS, and STOP-Bang may play a role in this screening.
Capone et al., 2013^38^	Case Control-PSG; Reiss and ABC scales	37 (9C) (28 DS)	19.8 (C) 21 (SD) years	5 years	– 86% of DS cases had OSA compared with 44% of controls; – Moderate-severe OSA was present in 54% of DS compared to 11% of controls; – Intermittent sleep-associated hypoxia and REM sleep deficits were also more frequent in DS. Across all subjects, prior tonsillectomy was not related to the presence or absence of OSA.	The results of the study suggest that OSA is a common comorbidity in adolescents and young people with DS and depression.

OSA: obstructive sleep apnea; DS: Down syndrome.

**Table 2:** Synthesis of articles selected for systematic review on prevalence, etiology, correlating factors, screening methods, and biomarkers for obstructive sleep apnea in Down syndrome patients (age<18 years).

Author/year	Study	N° of patients	Mean age (years/months)	Time of follow up	Outcome	Conclusion
Wijayaratne et al., 2021^48^	Case series-BMIZ score; sleep symptoms questionnaires	64 (DS)	3–19 years	-	Despite not being referred for clinical sleep assessment, 42% of children with DS recruited from the community had moderate/severe OSA.	There were no differences in the quality-of-life behavior, daytime functioning, and sleep symptom questionnaires although the clinical group had a higher body mass index (BMI Z score) and overt signs of obesity. These results highlight the importance of PSG screening in all children with DS.
Caloway et al., 2020^63^	Case series (Hypoglossal nerve stimulation-HGN)	20 (DS)	10-21 years	2 months	All 20 children were implanted with no long-term complications. We report two interval adverse events, both of which were corrected with revision surgery. Twenty participants completed the 2-month polysomnogram, with median percent reduction in titration AHI of 85% (interquartile range=75–92%). The median nightly usage for these children was 9.21 hours/night. There was a median change in the OSA-18 score of 1.15, indicating a moderate, yet significant, clinical change	HGN stimulation was safe and effective in the study population. Two minor surgical complications were corrected surgically. Overall, these data suggest that pediatric HGN stimulation appears to be a safe and effective therapy for children with DS and refractory severe OSA.
Lee et al., 2020^18^	Case series-PSG and FSIQ	30 (DS)	11.3 years	-	The presence of OSA in children with DS was 80% in the 6 to 18 age group, with 62.5% in the 6 to 12 age group; In individuals aged 6 and 12 years old, both OSA and% REM were associated with lower scores on the WPPSI-R Vocabulary test;	OSA can be highly prevalent in children with DS in the community. Among children with DS 6 and 12 years of age, OSA, and % REM were associated with their language function.
Waters et al., 2020^22^	Randomized Clinical TrialPSG	152 (DS)	5.0 (1st PSG) 8.2 (2nd PSG) years	3.5 years	In a tertiary sleep unit, a full spectrum of sleep-disordered breathing in Down syndrome was seen from infancy onwards. Children having only 1 study were more likely to have a normal or mild result than those having ≥2. Studies were more often severe in children age <2 compared to those ≥2 years. After age 2 years, OSA severity increased with age. Studies evaluating the effects of surgery (most often adenotonsillectomy) showed resolution of disease to mild or normal in 53.3%.	Children having only one study were more likely to have normal results. Children with multiple studies reflected disease surveillance, including follow-up after treatment interventions.
Nerfeldt et al., 2020^21^	PSG before and after OSA surgical treatment	138 (DS)	6.1 years	-	The prevalence of OSA was 82.6 and 39.9% had severe OSA (AHI: 7.6); comorbidities found were ear disease (60%), circulatory disease (51%) and endocrine disease (39%); 33 patients undergoing postoperative PSG had a residual prevalence of moderate or severe OSA of 63.6%; Pre and postoperative PSG of patients with ATE and APP presented median AHI changed from 21.1 to 12.4 and median OSA-18 from 54.0 to 35.0.	Uncertain surgical efficiency was indicated and no significant difference in results for ATE and APP was demonstrated. The authors point out that the frequency of PSG in the postoperative period was low and not systematic and that the groups were uneven and small.
Anand et al., 2021^30^	PSG, Child Behavior Checklist (CBCL), developmental quotient (DQ)	53 (DS)	<18 years	-	Of 53 subjects (three to 11.8 years), 51 (96%) were found to have obstructive sleep apnea (OSA). In both three to five year and six-to-12-year age groups, there was a statistically significant positive correlation between the CBCL scores and the AHI (rho=0.77 and 0.83, respectively). There was a statistically significant negative correlation between the DQ and the AHI (rho=-0.62). In multiple linear regression, AHI was the only independent variable that was associated with CBCL and DQ.	This study provides robust evidence that OSA can negatively influence the development and behavior in children with Down syndrome as in typically developing children. Moreover, with increasing severity of OSA, children with Down syndrome have more behavioral abnormalities, especially attention deficit and hyperactivity, and also have poorer development scores.
Chamseddin et al., 2019^45^	PSG	106 (DS)	2.0-18 years	6 years	90% of children had ≥1 medical comorbidities; 95 (90%) patients had OSA; and 46 (44%) had severe OSA. Mean SaO_2_ nadir was lower among obese than in nonobese children (80 vs 85%). Obese versus nonobese patients had a higher prevalence of severe OSA (56 vs 35%). The multivariable model showed that severe OSA was associated only with weight.	Obese children with DS are at a high risk for severe OSA, with weight as the sole risk factor. The results of this study show the importance of monitoring the weight of children with DS and counseling parents of children with DS about weight loss
Howard et al., 2020^61^	PSG and oAHI	24 (DS)	<18 years	5 years	There was no significant change in oAHI, oxyhemoglobin saturation nadir, ETCO_2_, or percent TST in REM after treatment for any treatment group. There was no association between reported symptoms and AHI severity or change in AHI.	In this cohort, the resolution of mild AOS was low for all treatment groups. These findings are consistent with the current understanding that OSA in children with DS is probably the result of multiple overlapping abnormalities contributing to the obstructive pathology
					OSA resolved in one patient treated with observation and two treated with medication, but worsened in two each in the medication and observation groups. Resolution of OSA occurred in 20% treated with medication, 7.7% with observation, and 0% with oxygen.	and suggests that a multimodal approach may be more appropriate in this population. Prospective studies will be useful in the future to establish a better understanding of treatment outcomes in children with DS and AOS lightweight.
Joyce et al., 2020^28^	Questionnaire Behavior rating inventory of executive functionpreschool version (BRIEF-P)	202 (DS)	36–71 months	-	OSA was associated with poorer working memory, emotional control and shifting.	Findings suggest that known executive function (EF) difficulties in DS are already evident at this young age. Children with DS already have limited cognitive reserve and cannot afford additional EF deficits associated with OSA. OSA is amenable to treatment and should be actively treated in these children to promote optimal cognitive development.
von Lukowicz et al., 2019^58^	Polygraphy	18 (DS)	6.3 years	1.5 year	Eighteen recordings had ≥3 hours of artefact free recording in both the pretreatment and posttreatment sleep study and were therefore included in the analysis. Mean age was 6.3 years; 83% had OSA prior to intervention. Mean OAHI was 6.4 before and 6.4 after the intervention; the DI3 and SpO2nadir also did not change. Only the DI90 decreased significantly from 2.7 to 2.1.	The 1-week intense myofunctional training camp evaluated here in children with DS had only a marginal effect on OSA. Whether a longer follow-up period or duration of intervention would yield stronger effects remains to be determined
Hill et al., 2018^50^	Case series-HPO	161 (DS)	0.5–6.0 years	-	In this training sample, the best HPO parameter predictors of OSA were the delta 12 s index >0.555 (sensitivity 92%, specificity 65%) and 3% oxyhemoglobin (SpO2) desaturation index (3% ODI)>6.15 dips/hour (sensitivity 92%, specificity 63%). Combining variables (delta 12 s index, 3% ODI, mean and minimum SpO_2_) achieved a sensitivity of 96% but reduced specificity to 52%.	HPO screening could halve the number of children with DS who require multichannel sleep studies and reduce the burden on children, families, and health services alike. This approach offers a practical universal screening approach for OSA in DS that is accessible to non-specialist pediatricians.
Beppler et al., 2018^52^	Case series-pediBand (prototype)	-	5 years	-	The potential of pediBand in measuring physiological signals that can be used in the diagnosis of OSA has been demonstrated.	It was demonstrated the potential of pediBand to successfully measure physiological signals that can be used in the diagnosis of OSA.
Best et al., 2018^60^	Retrospective case series	65 (DS)	4.8	8.5 years	The mean AHI was 10.7 events/hour after AT. Twenty-three patients (35.4%) underwent at least one additional surgical procedure after AT; 5 (7.7%) patients had ≥two additional procedures. The most common additional surgical procedures were revision adenoidectomies (n=8) and LT (n=13). Fifteen (23.1%) patients underwent at least one DISE to help direct selection of surgical site/s.	This retrospective case series provided the foundation for an algorithm for management of persistent OSA following primary AT in children with DS
Akkina et al., 2018^59^	PSG	24 (DS)	<18 years	3.5 years	The primary outcome was change in PSG parameters including AHI, OAHI, oxygen nadir, oxygen desaturation index, and mean carbon dioxide level. While improvement was seen in all PSG parameters, only improvement in oxygen nadir in children who had undergone prior AT was statistically significant (88.5 to 90.9%, p=04).	This study confirms a high proportion of multisite airway obstruction in DS patients with OSA. Although we observed an improvement across PSG measures, this study lacked power to detect statistically significant changes. DISE directed surgery holds promise as a beneficial tool for children with DS but a larger prospective study is needed before specific recommendations may be made on incorporating DISE into the OSA diagnostic and treatment algorithm for children with DS.
Slaats et al., 2018^65^	CT before the surgical procedure and PSG in the postoperative period	33 (DS)	4.3 years	3 years	Nineteen children underwent a second PSG after AT. Seventy-nine percent had persistent OSA (OAHI> 2 events/h). A greater than 50% decrease in OAHI was observed in 79% and these children had a significantly higher volume of the regions below the tonsils.	Children with severe OSA had a reduced air passage in the upper airway. Therefore, this study suggests that an image of the upper airway may have an influence on the choice of the text. This study is a pioneer in terms of analyzing the therapeutic response with CT analysis of upper airway.
Nehme et al., 2017^43^	Case series-PSG and sleep questionnaires	119 (DS)	6.6 years	10 years	Sleep-disordered breathing (SDB) was present in 42.9% of children, with its highest prevalence at age 8 years. Gastroesophageal reflux disease (GERD) was associated with lower odds of OAHI>5 events/hour; Presence of difficulty breathing at night, reported in the questionnaires of parents/caregivers, was significantly associated with apnea.	SDB is highly prevalent at all ages in children with Down syndrome. Symptoms did not predict SDB in this population, although GERD may mimic SDB.
Skotko et al., 2017^51^	Case series-PSG, Questionnaire, image exam	102 (DS)	3.0–24.0 years	6 months	The main outcome measure was the AHI. Using a Logic Learning Machine (with a questionnaire, imaging exam, and PSG) the best model had a cross-validated negative predictive value of 73% for mild OSA and 90% for moderate or severe OSA; positive predictive values were 55 and 25%, respectively.	In areas of the country where PSG is less available or affordable or when patients with DS are unable or unwilling to tolerate a sleep study, the model might offer, after validation, a viable alternative for providers looking to exclude moderate or severe OSA with a questionnaire.
Dudoignon et al., 2017^55^	Retrospective cohort	57 (DS)	5.9–6.2 years	5.5 years	33% patients required noninvasive respiratory support. Mean age at noninvasive respiratory support initiation was 7±7 years. On 11 patients with objective adherence data available, mean compliance at 2±1 years of treatment was excellent with an average use per night of 8hr46±3hr59 and 9 patient suing then on invasive respiratory support >4 hr/night. Non-invasive respiratory support was associated with an improvement of nocturnal gas exchange.	The study confirms the high prevalence and increased severity of OSA in children with DS. Upper airway surgery represents a first line treatment but has a limited efficacy. CPAP or NIV represent a very effective therapeutic option in case of persistent OSA after upper airway surgery. The major problem of CPAP/NIV is compliance but good results may be achieved by an experienced pediatric CPAP/NIV team.
Elsharkawi et al., 2017^53^	Urinary biomarkers	57 (DS)	4.0–9.1 years	-	Most night-sampled urinary biomarkers were elevated among individuals with DS relative to matched HC. No urinary biomarker levels differed between individuals with DS with vs. without OSA.	DS is associated with a different urinary biomarker profile when compared to HC. While urinary biomarkers may be predictive of OSA in the general pediatric population, a different approach is needed in interpreting urinary biomarker assays in individuals with DS.
Prosser et al., 2017^57^	PSG	21 (DS)	4.3–9.3 years	10 years	The median improvement in overall AHI and the OAHI were 5.1 events/hour and 5.3 events/hour (range, 22.9 to 41), respectively. The mean oxygen saturation nadir improved from 84 to 89%. The mean time with CO_2_>50 mmHg, central index, and percentage of rapid eye movement sleep were not significantly different. After surgery, the OAHI was <5 events/hour in 61.9% and ≤1 in 19% of patients.	In children with DS, persistent OSA after AT and lingual tonsil hypertrophy, LT significantly improved AHI, OAHI, and O_2_ saturation nadir. We recommend that children with DS should be evaluated for lingual tonsil hypertrophy if found to have persistent OSA following T&A.
Jayaratne et al., 2017^54^	Stereophotography 3dMDface	63 (DS)	4.86–7.49 years	-	Participants with DS had maxillomandibular hypoplasia with smaller	Anthropometric analysis of different craniofacial landmarks
					ear, nose, and eye measurements compared to neurotypically developing peers. We found no statistically significant differences in 3D photogrammetric measurements between participants with DS with or without OSA.	and measurements demonstrated that OSA cannot be correlated with the presence, absence, or degree of any of these structural alterations within this population
Hill et al., 2016^17^	Case series-Polygraphy	188 (DS)	0.6–6 years	-	Moderate or severe OSA, defined by an OAHI>5/hour, was found in 14%; and mild-moderate OSA (OAHI>1<5/h) in 59% of children. Male gender and habitual snoring predicted OSA but did not have independent predictive power in the presence of the other factors. Age in months, BMI, and tonsillar size did not predict OSA.	Moderate to severe OSA is common in very young children with DS. Examination of tonsillar size did not predict OSA severity. Population-based screening for OSA is recommended in these children and domiciliary cardiorespiratory polygraphy offers an acceptable screening approach. Further research is needed to understand the natural history, associated morbidity, optimal screening methodology, and treatment modality for OSA in these children.
Maris et al., 2016^46^	Case series-PSG and questionnaire to parents/caregivers	122 (DS)	4–18 years	5 years	The overall prevalence of OSA was 66.4%.	A significant inverse correlation was found between age and AHI
Maris et al., 2016^37^	DISE e PSG	41 (DS)	4.2 years	5.5 years	Adeno-/tonsillar obstruction was found in 75.6% of the patients, and these patients subsequently underwent UA surgery; A multilevel collapse was present in 85.4%. Tongue base obstruction was present in ten patients (24.4%) and epiglottic collapse in 48.8%; A significant improvement in oAHI from 11.4/h to 5.5/h was found, but persistent OSA was present in 52% of the children.	Most patients with DS and OSA present with multilevel collapse on DISE. Adenotonsillectomy results in a significant improvement of the oAHI; however more than half of the patients had persistent OSA, probably due to multilevel collapse.
Maris et al., 2017^44^	PSG	34 (DS)	2.7–5.8 years	5.5 years	The majority presented with severe OSA (58.9%). AT was performed in 22 children, tonsillectomy in 10 and adenoidectomy in two. Postoperatively, a significant improvement of the OAHI was measured from 11.4/hour to 3.6/hour, with a parallel increase of the minimum oxygen saturation. Children with initially more severe OSA had	AT results in a significant improvement of OSA in children with DS without a change in sleep efficiency or sleep stage distribution. Severe OSA was associated with a larger reduction of OSA severity.
					significantly more improvement after UA surgery. Persistent OSA was found in 47.1% of the children.	
Brockmann et al.; 2016^14^	Case Control-HPSG	44 (DS)	3.6 years	-	83% of individuals obtained HPSG results comparable to PSG; 61% of the study subjects had OSA, 18% of which were mild to moderate cases.	A portable polysomnographic home device may be helpful for diagnosing OSA in children with DS.
Diercks et al., 2016^62^	Hypoglossal nerve stimulator (HGN) Case report	1 (DS)	14 years	6 months	Hypoglossal nerve stimulator therapy was well tolerated and effective, resulting in significant improvement in the patient's OSA (overall AHI: 3.4 events/hour; AHI: 2.5–9.7 events/hour at optimal voltage settings depending on sleep stage and body position). Five months after implantation, the patient's tracheotomy was successfully removed and he continues to do well with nightly therapy.	The study demonstrated that the therapeutic measure obtained a well-tolerated and effective result, significantly reducing the patient's respiratory impairment.
Ono et al., 2015^33^	Case series-Questionnaire	90 (DS)	16.6 years	-	71% of the sample suffered from snoring, 59% had excitation, 25% apnea, and 22% nocturia; 24% had an unusual sleep posture, with the majority being from 6 to 15 years old (52%); Nocturia was the strongest predictor of unusual sleep positions for all OSA symptoms.	Symptoms related to OSA such as snoring and arousal are frequently observed in Japanese people with DS. Anatomical factors might contribute to the pathogenesis of OSA in people with DS, especially in the younger age groups. The high prevalence of unusual sleep postures may indicate a need to protect or compensate for OSA in people with DS who were less likely to be obese.
Brooks et al., 2015^26^	PSG, MSLT and neuropsychological tests	25 (DS)	7.2–18.7 years	1 year	The study demonstrated that the clinical findings were not predictive of the presence of OSA (PSG identified OSA in 10 out of 25). The author presented that there was no divergence in neuropsychological tests between children who had and did not have OSA.	Although SDB is common in children with DS, it is not a major contributor to their cognitive deficits. Cognitive function is related to the amount of sleep and particularly slow wave sleep. Successful treatment of SDB may improve their attention.
Thottam et al., 2015^39^	PSG in the pre and postoperative period of AT	36 (DS)	9.0 years	5.5 years	Children with DS who underwent surgery showed significant reductions in PSG obstructive and central AHI; 86.7% of children with DS presented a significant reduction in AHI for moderate or mild disease and 66.7% had resolution of central sleep apnea in the postoperative period.	Children with DS who underwent AT demonstrated significant reductions in both obstructive and central apneic indices on PSG. A significant number of patients with central sleep apnea demonstrated resolution postoperatively.
Coverstone et al.; 2014^15^	PSG and McGill oximetry score	119 (DS)	7.0 years	3.5 years	OAHI was ≥2.5 for 50% of all individuals; 36.1% had McGill equal to 2 and 14.3% equal to 3 or 4; McGill oximetry scores 3 and 4 are related to OSA and indicate clinical follow-up.	McGill oximetry scores of 3 or 4 reliably identified patients with marked OSDB. The possibility of central apneas causing hypoxemia must be considered in those with McGill Score 2.
Lin et al., 2014^19^	Case series-PSG and McGill oximetry scale	49 (C) 49 (DS)	6.3 (C) 6.2 (DS) years	-	34.69% of children with DS presented OSA; OSA in children with DS was more severe than in children in normal development; Children with DS had a higher mean of pCO_2_ during sleep and worse scores on McGill oximetry.	Children with DS have more complicated OSA and more impaired gas exchange compared to children in the control group, with similar symptoms.
Breslin et al., 2014^27^	Case series-PSG and cognitive assessment	38 (DS)	9.7 years	3 months	Among children with DS, mean verbal IQ score was 9 points lower in those with comorbid OSA (AHI>1.5) than in those without OSA, and performance on measures of cognitive flexibility was poorer. Children with OSA showed increased light-stage sleep at the expense of slow-wave sleep.	The results suggest that more work is needed to understand the influence of poor sleep on learning in DS and other neurodevelopmental syndromes, many of which demonstrate disordered sleep to some extent.
Stores et al., 2014^47^	Case series-Questionnaire, Oximetry	31 (DS)	2.3–16.7 years	-	No significant association was found between objective measures of restlessness during sleep and ‘snoring’, nor were objective measures of restlessness related to reductions in overnight blood oxygen levels. –The objective measure of snoring was significantly associated with reductions in overnight blood oxygen levels.	The overnight measures used in the present study proved feasible and largely acceptable to the children and their families. More time spent familiarizing children with the procedure and the use of more recently developed recording systems would be likely to improve the success rate with this particular procedure.
Austeng et al., 2014^40^	Case series-PSG	29 (DS)	8.0 years	-	AHI>1.5 in 28 of 29 children and an OAI>1 in 24 of 29 children. 19 children (66%) had an AHI>5 and 17 children (59%) had an OAI>5 which indicated moderate to severe OSA. No correlation was found between OSA and obesity or gender.	The high prevalence of disease found in these previously undiagnosed 8-year-old children underlines the importance of performing OSA diagnostics in children with DS throughout childhood. These findings suggest that the prevalence of OSA remains high up to early school years.

OSA: obstructive sleep apnea; DS: Down syndrome.

**Table 3:** Synthesis of articles selected for systematic review about other sleep-related problems in Down syndrome (other than obstructive sleep apnea); (age>18 years).

Author/year	Study	N° of patients	Mean of age (years/months)	Time of follow up	Outcome	Conclusion
Gomes et al., 2020^25^	Case series-maximum mouth opening-MMO; maximum bite force-MBF; maximum voluntary clench-MVC	35 (C) 35 (DS)	19–40 years	-	Electrical activities of the masseter and temporal muscles (at rest and in maximum voluntary clench-MVC), maximum bite force-MBF, and maximum mouth opening-MMO were investigated.	Masseter and temporal muscle hypotonia were found in all atypical subjects with DS. This muscle dysfunction strongly was related to overweight/obesity, risks for development of cardiovascular/metabolic diseases, OSA severity, successive snoring episodes, and salivary flow reduction in DS.

OSA: obstructive sleep apnea; DS: Down syndrome.

**Table 4:** Synthesis of articles selected for systematic review about other sleep-related problems in Down syndrome (other than obstructive sleep apnea); (age<18 years).

Author/Year	Study	N° of patients	Mean age (years/months)	Time of follow up	Outcome	Conclusion
Chawla et al., 2021^24^	Case series-CSHQ and sleep clinic	76 (DS)	-	-	The first study to report the prevalence of sleep problems in Australian children with DS and to compare a community and referred group of children with DS directly.	This study reports a high prevalence of sleep problems in both a community and referred group of Australian children with DS, and suggests that there are many children with DS and sleep problems, particularly non-respiratory difficulties, who are potentially not receiving adequate treatment.
Santoro et al., 2021^34^	PSG	82 (DS)	<18 years	-	Reported sleep positions were skewed towards lateral/decubitus (82.9%) compared to prone (11.0%) and supine (6.1%). This was consistent with hypnogram data where 71% of total sleep time in lateral/decubitus positions compared to prone (13%) and supine (6%). Tonsillectomy was associated with lower obstructive AHI (OAHI) Sleep position was not associated with age, gender, race, ethnicity, nor history of tonsillectomy. Preferred sleep position was not correlated with OAHI or OSA severity.	This study highlights the possibility that children with DS may have preferential sleep positions that cater to optimized airflow in the context of OSA, although further prospective study is needed.
Shaw et al., 2021^29^	DSM 5 criteria and individual medical record numbers (MRN's) Chi-square test and Fisher's exact AND Student's *t*-test	370 (DS)	2–17 years	1.5 year	Compared to typically developing children, children with DS may have more challenges with adaptive functioning in the school setting (examples include complying with directions and task persistence). Parents and teachers report higher rates of	Developmental/behavioral assessment is integral for detection of co-morbid conditions among a pediatric DS population and prevention of diagnostic overshadowing.
					non-compliance in children with DS compared to those without DS secondary to their executive functioning and adaptive deficits	
Bassam et al., 2021^31^	Heart rate (HR) and pulse transit time (PTT) (a surrogate inverse measure of BP change)	19 (DS) 19 (C)	3–18 years	-	Children with DS exhibited reduced nocturnal dipping of HR during total sleep. Fewer children with DS exhibited a greater than 10% fall in HR between wake and REM sleep compared to TD+children.	Findings demonstrate significantly reduced nocturnal dipping of HR in children with DS compared to TD children matched for SDB severity, suggesting SDB has a greater cardiovascular effect in these children. Further studies are required to fully understand the mechanisms involved and to assess if treatment of SDB improves nocturnal dipping.
Siriwardhana et al., 2021^32^	PSG, nasal pressure, and transcutaneous carbon dioxide (TcCO_2_)	14 (DS) 14 (C)	3–19 years	2.5 years	Children with Down syndrome also had significantly lower average oxygen saturation associated within each analysis window compared to typically developing children	Higher loop gain in children with Down syndrome and sleep disordered breathing indicates that these children have more unstable ventilatory control, compared to age, gender and sleep disordered breathing severity matched typically developing children. This may be due to an inherent impairment in ventilatory control in children with Down syndrome contributing to their increased risk of sleep disordered breathing, which may inform alternative treatment options for this population.
Richard et al., 2020^42^	Case series-PSG and clinical files	28 (DS) 28 (C)	<18 years	5 years	Mean transcutaneous partial pressure of carbon dioxide (PtcCO2) during sleep was significantly higher in patients with DS compared to controls.	This was the first study to compare nocturnal gas exchange in children with DS to a control group of children with similar OSA, but not DS. Data demonstrated that children with DS have increased transcutaneous partial pressure of carbon dioxide (PtcCO_2_) regardless of the presence of OSA and its severity. This may be due to respiratory muscle hypotonia and/or ventilatory control alteration in patients with DS.
Giménez et al., 2018^16^	Case series-PSG, self-reports and, actigraphy.	35 (C) 47 (DS)	39.2 (C) 39.6(DS) years	-	Adults with DS had lower sleep efficiency, lower %REM, higher prevalence of OSA (78 *versus* 14%) and a higher AHI than patients in the control group. The DS group questionnaires (PSQI and ESS) did not reflect the sleep disorders detected in the PSG.	Adults with DS have more sleep disorders, especially OSA. Sleep disorders were not detected by self-reported sleep measures. Actigraphy, PSG and simplified devices validated for OSA screening are important tools for diagnosis.
Maris et al., 2016^20^	Case series-CSHQ and PSG	54 (DS)	8.9 (C) 7.5 (DS) years	-	According to the CSHQ, 74.1% of children with DS had sleep problems.	Children with DS have a significantly higher prevalence of sleep problems, compared to.
					The general sleep problems were not related to age or gender, however, boys suffer more from daytime sleepiness. Symptoms of respiratory sleep disorders are related to parasomnias, longer sleep duration, and more daytime sleepiness.	normal developing healthy school-aged children. No correlations were found between the relative reports on sleep problems and the underlying OSA or severity of OSA.
Ong et al., 2018^56^	Case series-Retrospective cohort database analysis	51292 (DS)	0–20	15 years	Tonsillectomy with adenoidectomy was the most common procedure in both groups, but the proportion of tonsillectomy with adenoidectomy decreased over time. –The proportion of palatal surgery and tracheostomy also decreased significantly, whereas there was an increase in the proportion of lingual tonsillectomies, tongue-base reduction procedures, and supraglossoplasty performed in both groups over time. The relative rates of change in these procedures were higher in the DS population.	Tonsillectomy with adenoidectomy remains the most commonly performed procedure, although there was a significant increase in other sleep surgeries performed (LT, tongue-base reduction, and supraglossoplasty) between the two study periods, especially in children with DS.
Mylavarapu et al., 2016^64^	Computational fluid dynamics, virtual surgery, CT and MRI	10 (DS)	5 years	-	There was a reduction, in 8 out of 10 patients, of AHI and the resistance of upper airway, when compared to baseline values.	This study highlights the need for future studies, before using this technique in surgical plans.
Hoffmire et al., 2014^23^	Case series-CSHQ and PSQ	107 (DS)	7–17 years	2 years	65% of children with DS had sleep problems in the CSHQ, but these problems were not reported by their parents; In PSQ, 46% of children had sleep-related breathing problems and 21% sleep-related movement disorders; Children with asthma, autism and a history of enlarged adenoids and tonsils had more frequent sleep problems than children without these comorbidities.	Sleep disorders are important but also under-recognized problems in children with DS. It appears to be correlated with some prevalent comorbidities, which may provide guidance to augment current practice guidelines to evaluate sleep problems in this population.
Nisbet et al., 2014^36^	Case series-PSG and body posture record during sleep	76 (C) 76 (DS)	5.1 (C) 4.6 (DS) years	4.5 years	Sensor-recorded position (supine, prone, lateral) was expressed as the percentage of total sleep time. The apnea-hypopnea index (AHI) was calculated in each sleep state (NREM, REM), position, and position-sleep state combination. AHI was higher in REM than NREM; nonetheless, the NREM AHI was higher in DS than NREM AHI that controls.	In DS and non-DS children alike, respiratory events are predominantly REM-related. However, when matched for OSA severity, children with DS have a higher NREM AHI, which is worse in the supine position, perhaps indicating a positional effect compounded by underlying hypotonia inherent to DS.
					The percentage of prone sleep was greater in DS than controls, but of supine or non-supine sleep was not different between them.	
Konstantinopoulou et al., 2016^41^	Case series-PSG, ECG and BPN	23 (DS)	2.7 months	-	At four months, there were no changes in cardiovascular outcomes or sleepiness between those on actual versus sham CPAP. Hours of actual CPAP use were associated with improved left ventricle function.	In children with DS, left ventricle diastolic function correlated with OSA severity, which improved with the use of CPAP. There was a tendency towards increased sleepiness in those with OSA, which correlated with the rate of awakening.
Senthilvel and Krishna, 2011^35^	PSG	17 (C) 17 (DS)	6 years	1.5 year	History of previous tonsillectomy (41%), congenital heart disease (of any type) (82%) and hypothyroidism (41%) of SD compared to 24%, 12 and 0%, respectively of C. SD assumed a unique body position sitting cross-legged flopped-forward with head resting on bed while asleep.	Some DS children assume a peculiar body position, sitting cross-legged flopped-forward with head resting on bed while asleep. This is absent in age and gender-matched controls showing otherwise similar PSG characteristics. The reason for this posture is unclear from this study.

OSA: obstructive sleep apnea; DS: Down syndrome.

## DISCUSSION

### Prevalence, etiology, and correlating factors for sleep disorders in individuals with Down syndrome

The main sleep disorder associated with DS in the selected articles was obstructive sleep apnea (OSA), with a prevalence ranging from 60 to 95%, depending on the criteria used for diagnosis and the age of the patients. However, the heterogeneity between the studies in terms of the method used for the diagnosis of the respiratory disorder is noteworthy: polysomnography (PSG), home polysomnography (HPSG), home night sleep records, cardiorespiratory polygraphy, housekeeping, McGill oximetry score, and actigraphy. In some cases, only questionnaires or scales were used, such as: Pittsburgh Sleep Quality Index (PSQI); Epworth Sleepiness Scale (ESS); Berlin Questionnaire (BQ); Child Sleep Habits Questionnaire (CSHQ), which may have compromised the assessment of prevalence ^14–22^.

We found few studies of parasomnia, insomnia, and daytime sleepiness in individuals with DS. Two studies found that some sleep problems were significantly more common in the population with DS, such as: resistance to bedtime, sleep duration, sleep anxiety, night watch, parasomnias, and daytime sleepiness^20,23,24^. However, none of the studies addressed the presence of parasomnias and their most frequent types isolated.

Maris et al. studied the occurrence of parasomnias, insomnia, and daytime sleepiness by comparing two groups of DS patients, the first with younger individuals (4 to 6.9 years) and the second with older children (over 11 years). Parasomnia was reported significantly less frequently with increasing age, which is also seen in normally developed children. In children with DS, in contrast to children with normal development, a decrease in the prevalence of sleep anxiety with increasing age was observed. Delay in falling asleep occurred more frequently in children with DS than children with normal development. Sleep onset delay in DS was significantly more common with increasing age and in children with sleep anxiety. Daytime sleepiness occurred more frequently among boys, regardless of age^20^.

Gomes et al. examined the electrical activities of the masseter and temporal muscles in patients with DS. These activities are atypical in these patients, indicating that DS patients are at greater risk for overweight/obesity, cardiovascular/metabolic diseases, OSA severity, and a salivary flow reduction ^25^.

### Sleep disordered breathing in patients with Down syndrome and its negatives effects on cognitive function

Several studies have been done associating sleep parameters and cognitive functions. In one interesting study, neurophysiological parameters obtained in the PSG and multiple sleep latency test (MSLT) were correlated with the answers in cognitive tests, and found that shorter total sleep duration and greater sleepiness were associated with poorer cognitive function in patients with DS. Furthermore, the lowest percentage of slow-wave sleep was found to be a predictor of better adaptive behavior and academic performance in individuals with DS. Another important finding was that appropriate treatment of sleep-disordered breathing in DS patients resulted in better cognitive performance, especially in the area of attention^26^.

Lee et al. compared the results of PSG studies and cognitive scales assessing language, behavior, and intellectual performance in patients with DS. They found that reduction in the percentage of REM sleep and the presence of OSA were associated with impaired language function in patients with DS^18^. Other studies with similar designs have correlated a reduction in slow-wave sleep with poorer performance in verbal learning and executive functions in patients with DS^26–30^.

In addition, children with DS are at higher risk for sleep disordered breathing (SDB), which can negatively affect the cardiovascular system. Besides, the risk of future cardiovascular events is increased in these patients due to decreased nocturnal reduction in heart rate (HR) and blood pressure (BP)^31^.

Another study discussed the unstable ventilatory control that is more common in children with DS. This finding indicates that these children are at greater risk for sleep disordered breathing than patients without DS^32^.

### Sleep related movement disorders and unusual sleep postures in Down syndrome patients

Sleep problems in children with DS go beyond OSA and other sleep-disordered breathing. Sleep-related movement disorders are also more common in individuals with DS^20,23^. Hoffmire et al. observed that 21% of children with DS were positive for sleep-related movement disorders measured with the CSHQ. Also, this risk was associated with asthma, autism, and a history of enlarged adenoids and tonsils^23^.

Other previous studies applied questionnaires and found that atypical positions such as leaning forward with legs back, leaning forward with legs forward, leaning forward with legs crossed, and sitting were common and were often related to the presence of OSA diagnosis^33^. Additionally, patients with DS commonly present the unique position of sitting with a flopped-forward body in which the head rests on the bed while asleep, which contributes to optimized airflow^34^. The reason for this position is unclear, but authors conjectured that this may be a protective mechanism for airway patency^35^.

Another study used PSG and recording of body positions during sleep using sensors. Subjects with DS spent a significantly longer duration of sleep in the prone position and less in the right lateral decubitus position compared to subjects without the paired syndrome by age and sex^36^.

## OBSTRUCTIVE SLEEP APNEA IS THE MOST PREVALENT SLEEP DISORDER IN PATIENTS WITH DS

As previously mentioned, OSA is the most prevalent sleep disorder in these patients, and there are a few reasons for this. Maris et al. found that children with DS have anatomical narrowing of the upper airway at different levels and are more prone to collapse and thus at higher risk for OSA. Other factors contribute to explain the association between OSA and DS such as muscle hypotonia, higher incidence of congenital heart disease, hypothyroidism, lung disease, immunodeficiency, relative macroglossia (due to smaller bone framework of mandible and maxilla)^37^.

Some studies have included a control group of children and adolescents without DS and found a prevalence of less than 20% of OSA, highlighting the important association between DS and OSA^16,21,22,38,39^. Some authors claim that individuals with DS have more severe OSA and greater refractoriness to treatment^16,19,39,40^.

According to studies by Konstantinopoulou et al., left ventricle diastolic function correlates with the severity of OSA, which improves with the use of continuous positive airway pressure (CPAP). In addition, they noted a tendency for increased sleepiness in individuals with OSA, which was correlated with the awakening index. Further studies are needed to confirm the findings described^41^.

Coverstone et al. evaluated the probability of developing OSA in DS patients with pulse oximetry and classified them according to the McGill score. Patients with McGill score 3 or 4 (more than 3 desaturations below 80–85% in one night of sleep) or McGill score 2 with increased body mass index (BMI>25 kg/m^2^) were referred by an otorhinolaryngologist due to their increased risk of adenotonsillar hypertrophy. The authors suggest that patients with low McGill scores should be monitored regularly by a specialist to obtain continuous assessment^15^.

Nicolas et al. conducted the first study to compare nocturnal gas exchange in children with DS with a control group of children with similar OSA. They concluded that patients with DS have respiratory muscle hypotonia and/or an alteration in ventilatory control^42^.

Nisbet's study showed that children with DS and OSA had a similar dominance of rapid eye movements (REM) in breathing events compared to children with OSA and without DS, but the children with DS had a higher NREM apnea-hypopnea index (AHI), even though they were similar in terms of total AHI and had a similar percentage of sleep time in NREM. Notably, children with DS in supine position had a higher NREM AHI than in the non-supine position^36^.

### Obesity and other possible predictive variables for obstructive sleep apnea in patients with Down syndrome

The association between obesity and the occurrence or severity of OSA in patients with DS is controversial. Most studies included that no correlation exists between higher BMI and OSA in this population^14,17,19,33,40,43,44^, but it should be noted that most of these studies included children only.

On the other hand, Chamseddin et al. correlated obesity not only with a higher occurrence of OSA in DS patients, but also with a high severity of OSA^45^. Similarly, two other studies reported that patients with DS, who had high BMI and/or hypothyroidism, had greater upper airways narrowing and consequently a higher severity of OSA. They also highlight the importance of preventing obesity in adolescence to reduce the incidence of OSA in adults with the syndrome^15,16^. Therefore, there is no consensus among researchers on the relationship between OSA and overweight/obesity.

There are some predictive variables for the occurrence of OSA in patients with DS, such as presence of parasomnias, longer total sleep time, daytime sleepiness, snoring, witnessed apnea and nocturia^18,23,27,33,43,46^. Hoffmire et al. described that the presence of asthma or allergic rhinitis is not related to an increased risk of OSA in patients with DS^23^. In addition, there is no consensus among researchers on the association between gastroesophageal reflux disease (GERD) and OSA in this population^23,43^. Nehme et al. pointed out that the symptoms of GERD may be similar to those of OSA, leading to a better performance of the PSG exam, which could contribute to a greater identification of OSA in these patients^43^.

### Screening methods and biomarkers for obstructive sleep apnea in patients with Down syndrome

Although PSG is considered the gold standard examination to define OSA, screening methods have been investigated to evaluate sleep disorders in this population. Considering the technical difficulties in performing PSG, the lack of availability of the exam, and its high cost, alternatives must be sought. In this manuscript, it was shown that only about 50% of selected studies used PSG to define OAS in DS patients. Although the presence of restlessness and snoring are important indicators of OSA in patients with DS, no significant association between these indicators and low oxygen saturation was found in the Stores et al. study. Therefore, the authors suggest that the presence of restlessness may be an important clinical feature to assess the need for a PSG^47,48^. Questionnaires and clinical and laboratory data are used to identify moderate to severe OSA in this population^49^.

Another alternative is screening by home pulse oximetry (HPO), which could halve the number of children with DS who need multichannel sleep studies^50^. Although these tests are useful, they cannot be used in isolation to diagnose breathing-related sleep disorders^47,50^.

Two studies have used questionnaires as a tool for diagnosing OSA. In the first study, conducted by Hoffmire et al., the CSHQ and the Pediatric Sleep Questionnaire (PSQ) were applied^23^. In the second study, conducted by Maris et al., the CSHQ and the PSG were used as auxiliary tools for diagnosis^20^. Both studies concluded that a large number of children with DS had sleep behavior disorders (insomnia, parasomnias) and sleep-related breathing problems, but curiously, their caregivers did not complain of such conditions. No relationship was found between the scores obtained in the CSHQ and the OSA index^20,23^. Therefore, the isolated use of questionnaires as a screening tool for OSA does not seem to be an effective method.

In an interesting study conducted at Boston Children's Hospital, a predictive model was created to help screen for OSA in patients with DS. The variables used were age, sex, race, height, weight, BMI, sedentary behavior, blood pressure, peripheral O_2_ saturation, neck circumference, macroglossia assessment, Mallampatti classification, Friedman/Brodksy scores, classification of scores, and current treatment for asthma, GERD, or thyroid disease. Results of the following scales and questionnaires were also used: PSQ, CSHQ, and Sleep Disorders Scale (SRBD), which were applied to parents and/or guardians. Using a logic learning machine, the best model had a validated negative predictive value of 73% for mild OSA and 90% for moderate or severe OSA. The final model revealed that the most relevant variables (out of 101) were certain CSHQ questions, SRBD questions, and the hypertension percentile. The study shows promising results with models using clinical data and questionnaires and may be an interesting tool for screening OSA in patients with DS^51^.

Similarly, Beppler et al. have developed a prototype called PediBand to help diagnose OSA in patients with DS. PediBand assesses the following physiological parameters: heart rate and its variability, respiratory rate, and O_2_ saturation. This model is a promising tool to investigate sleep disordered breathing in DS. However, as it is still a prototype, further clinical studies are needed to strengthen the evidence for its use^52^.

OSA biomarkers have also been studied in individuals with DS. Elsharkawi et al. measured biomarkers such as epinephrine, norepinephrine, dopamine, serotonin, glycine, taurine, γ-aminobutyric acid (GABA), glutamate, phenylethylamine (PEA), aspartic acid, histamine, 3,4-dihydroxyphenylacetic acid (DOPAC), 5 hydroxy acid (5-HIAA), tyramine, and tryptamine in DS patients with OSA, DS patients without OSA, and in healthy controls, which were equal in age and gender. The results showed that epinephrine, norepinephrine, dopamine, and taurine were good predictors of the presence or absence of DS, but these results were not statistically significant in distinguishing the presence or absence of OSA in these patients. Thus, these urine biomarkers were ineffective tools for screening OSA in individuals with DS^53^. It should also be noted that the low availability of the tests and the technical difficulties in performing them are major obstacles to its use in clinical practice.

Jayaratne et al. conducted a 3D comparison of patients with and without OSA. An anthropometric analysis scheme was developed to quantify facial norms with well-defined reference points focusing on the soft tissues of the external morphology. Most anthropometric measures were lower in individuals with DS, indicating maxillomandibular hypoplasia and reduced measures of the nose, ears, and eyes. However, the authors compared patients with DS and OSA versus patients with DS and without OSA and found no significant differences in these measures. A limiting factor was the restriction to ethnicity (Caucasians only), which requires a more in-depth analysis of different ethnicities and a wider age range^54^.

### Treatment options for obstructive sleep apnea in patients with Down syndrome and new perspectives

The main treatment options for OSA in DS patients are CPAP, surgery, and weight control. Several therapeutic alternatives have been studied, considering that CPAP therapy is not always available or tolerated, that surgical intervention is not always appropriate, and that there is no consensus on whether there is a direct relationship between obesity and OSA in patients with DS.

Several studies indicate that adenotonsillectomy (AT) is still the gold standard for the treatment of OSA in patients with DS^37,39,44,55–58^. Other possible interventions include lingual tonsillectomy (LT) and supraglossoplasty (SGP). LT may be considered in the context of residual OSA after AT, despite its lower efficacy^56^. The authors emphasize the importance of surgical planning with the identification of upper airway obstruction sites and the main tool for this purpose is drug-induced sleep endoscopy (DISE)^20,59,60^.

Concerning drug treatment, further studies are needed to clarify its role in the OSA in patients with DS. Intranasal corticosteroids may contribute to a local anti-inflammatory effect by reducing apnea, but the effectiveness has not been fully demonstrated. A retrospective study showed that children who underwent AT and used nasal corticosteroids had less residual OSA than children who did not undergo this drug treatment. Considering the small sample size of the study, the role of medication in the treatment of residual OSA in DS remains uncertain^60,61^.

One of the new interventions that have been studied is myofunctional orofacial training (MT). MT is based on the principle of strengthening orofacial and cervical functions for muscular balance, thereby reducing the chances of recurrences due to the maintenance of inadequate functional patterns^58^. Diercks et al., on the other hand, pioneered the investigation of a hypoglossal nerve stimulation implant in the pediatric range as a prospect for treating a patient with DS associated with severe OSA. The study demonstrated that the therapeutic intervention produced a well-tolerated and effective outcome and significantly reduced the patient's respiratory impairment^62,63^.

Three-dimensional reconstruction models from imaging exams — such as computer tomography (CT) and magnetic resonance imaging (MRI) — look promising, but studies with a larger sample of patients are needed to verify their real effectiveness^64,65^.

In conclusion, individuals with DS are at high risk of developing sleep-related breathing disorders, mainly due to anatomical changes in the upper airway. The presence of sleep disorders contributes to the deterioration of cognitive function in patients with DS. PSG is the gold standard exam for determining OSA, but the high cost and difficulty of technical approach are pushing for better options. OSA is the most studied sleep disorder in patients with DS and its main treatment is AT. There are some emerging perspectives on OSA treatment in patients with DS, but high-quality trials of multimodal interventions are needed to provide robust evidence for the treatment of OSA in DS individuals.
